# Supplementary material for: Glycerol-weighted chemical exchange saturation transfer nanoprobes allow 19F/1H dual-modality magnetic resonance imaging-guided cancer radiotherapy
Source: Nat Commun. 2023 Oct 20;14:6644. doi: 10.1038/s41467-023-42286-3 (PMC10589257; doi:10.1038/s41467-023-42286-3)
Supplement: Supplementary file 1 — Supplementary Information [file 41467_2023_42286_MOESM1_ESM.pdf]

Supplementary information

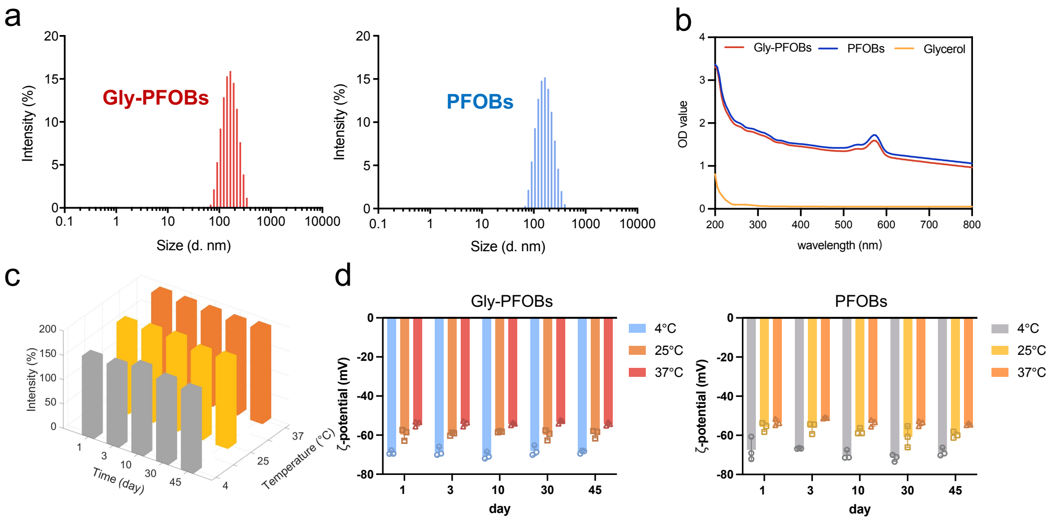

Supplementary Figure 1. Characteristics of Gly-PFOBs and PFOBs. a. Hydrodynamic particle sizes of Gly-PFOBs and control probe without CEST signals (PFOBs) were characterized by DLS. Repeated 4 times independently with similar results. b. Corresponding UV-VIS spectra of glycerol, Gly-PFOBs, and PFOBs. Repeated 4 times independently with similar results. c. Measurement of changes in the intensity-averaged mean particle size (hydrodynamic particle diameter) of PFOBs (n=5 independent measurements). Three-dimensional (3D) graphs were generated using Matlab based on the average values obtained. d Measurement of changes in the zeta potential of CESTs and PFOBs at 4 °C, 25 °C, and 37 °C within 45 days (n=3 independent measurements).

Duration	*Gly-PFOBs dialyzed against ultra-pure water(μmol)*	*Ultra-pure water(μmol)*	*Gly-PFOBs dialyzed against saline (μmol)*	*Saline (μmol)*
1 h	3099 ± 0.59	16.04 ± 0.59	3098 ± 0.84	16.74 ± 0.84
2 h	3094 ± 0.38	21.43 ± 0.38	3086 ± 0.87	29.14 ± 0.87
4 h	3090 ± 0.86	25.23 ± 0.86	3078 ± 1.8	37.16 ± 1.80
6 h	3083 ± 0.39	32.30 ± 0.39	3074 ± 3.3	41.43 ± 3.25

Supplementary Table 1. The glycerol content in Gly-PFOBs and in different dialysates (ultra-pure water or saline) at different time points were determined by Free Glycerol Assay Kit (abcam), n=3 independent samples.

Duration	*Gly-PFOBs without dialysis*	*Gly-PFOBs dialyzed against Ultra-pure water (mmol/L)*	*Gly-PFOBs dialyzed against Saline (mmol/L)*
1 h	623.51 ± 39.42	622.93 ± 12.18	572.44 ± 33.97
2 h		615.39 ± 4.87	571.62 ± 12.72
4 h		614.64 ± 18.42	562.21 ± 20.13
6 h		612.96 ± 21.5	561.51 ± 15.83

Supplementary Table 2. The glycerol concentration in Gly-PFOBs without dialysis and glycerol concentration in Gly-PFOBs dialyzed against different dialysates (ultra-pure water or saline) at different time points were determined by Gas Chromatography/Mass Spectrometry (GC/MS) analysis, n=3 independent samples.

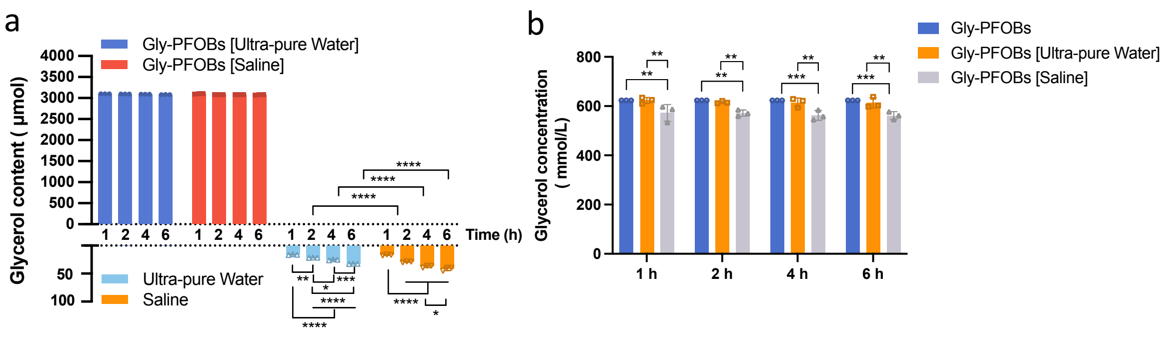

Supplementary Figure 2. Evaluation of the persistence of glycerol component on the Gly-PFOBs. a. The glycerol content in Gly-PFOBs and different dialysate (ultra-pure water or saline) at different time points was determined by Free Glycerol Assay Kit (abcam), * *P* =0.0242, glycerol in ultra pure water (dialysate) 2 h *vs.* 4 h; * *P* =0.0110, glycerol in saline 4 h *vs.* 6 h, ** *P* =0.0016, glycerol in ultra pure water (dialysate) 1 h *vs.* 2 h, *** *P* =0.0001, glycerol in ultra pure water (dialysate) 4 h *vs.* 6 h, **** *P* < 0.0001, two-way ANOVA, Tukey's multiple comparisons test; b. Gas Chromatography-Mass Spectrometry (GC-MS) analysis was performed to determine glycerol concentrations in Gly-PFOBs after different duration of dialysis (1 h, 2 h, 4 h, 6 h). ** *P* =0.0017, Gly-PFOBs 1 h *vs.* Gly-PFOBs [Saline] 1 h, ** *P* =0.0014, Gly-PFOBs [Ultra-pure water] 1 h *vs.* Gly-PFOBs [Saline] 1 h; ** *P* =0.0061, Gly-PFOBs 2 h *vs.* Gly-PFOBs [Saline] 2 h, ** *P* =0.0017, Gly-PFOBs [Ultra-pure water] 2 h *vs.* Gly-PFOBs [Saline] 2 h; *** *P* =0.0002, Gly-PFOBs 4 h *vs.* Gly-PFOBs [Saline] 4 h, ** *P* =0.0011, Gly-PFOBs [Ultra-pure water] 4 h *vs.* Gly-PFOBs [Saline] 4 h; *** *P* =0.0002, Gly-PFOBs 6 h *vs.* Gly-PFOBs [Saline] 6 h, ** *P* =0.0014, Gly-PFOBs [Ultra-pure water] 6 h *vs.* Gly-PFOBs [Saline] 6 h, two-way ANOVA, Tukey's multiple comparisons test. Data are presented as mean ± standard deviation (SD) (n = 3 independent samples).

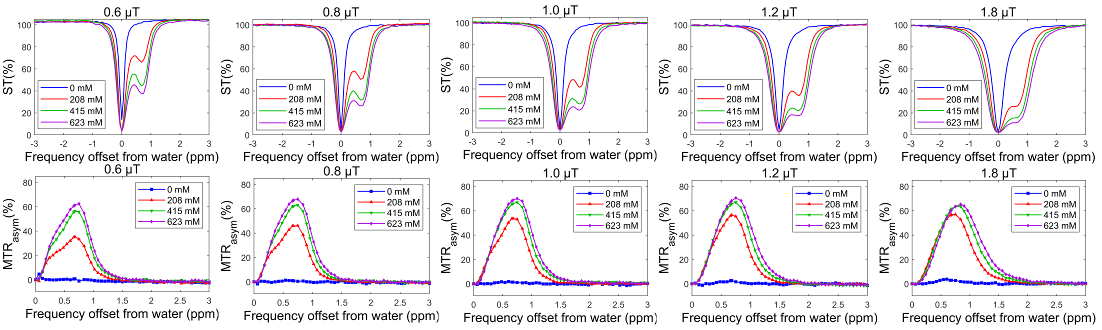

Supplementary Figure 3. CEST signal properties of Gly-PFOBs. Z-Spectra and MTR_{asym}(%) curve of Gly-PFOBs containing different concentrations (0 mM, 208 mM, 425 mM, and 623 mM) of glycerol at different saturation pulse powers (0.6 μT, 0.8 μT, 1.0 μT, 1.2 μT, and 1.8 μT) with same saturation pulse duration (5s).The experiment was repeated n=3 times with similar results.

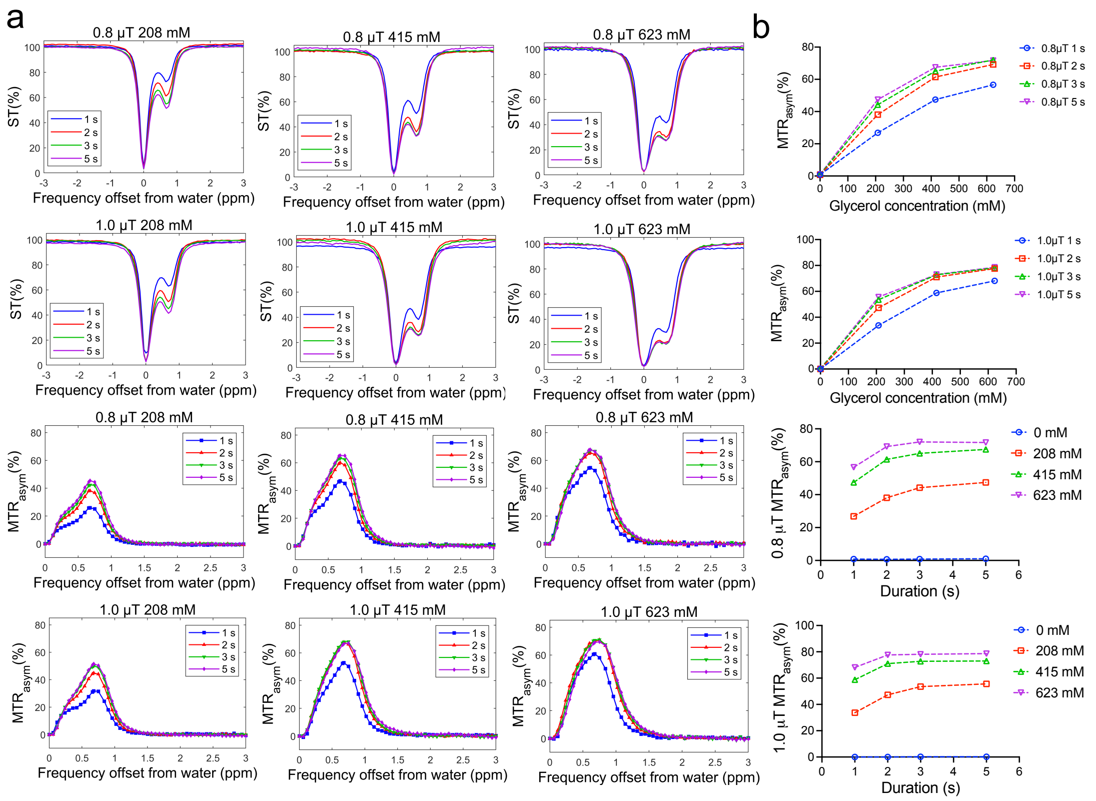

Supplementary Figure 4. CEST signal properties of Gly-PFOBs. a. Z-Spectra and MTR_{asym} curve of Gly-PFOBs containing different concentrations (0 mM, 208 mM, 425 mM, and 623 mM) of glycerol at different saturation pulse durations (1 s, 2 s, 3 s, and 5 s) and different saturation pulse powers (0.8 μT and 1.0 μT), and b. corresponding statistical analysis. The experiment was repeated n=3 times with similar results.

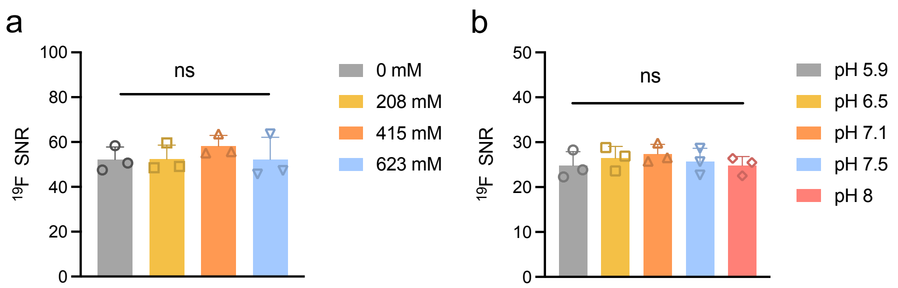

Supplementary Figure 5. Statistical results of ^{19}F-MRI signal-to-noise ratio (SNR) of the phantom. a. ^{19}F-MRI SNR statistical results of Gly-PFOBs with different glycerol concentrations b. ^{19}F-MRI SNR statistical results of Gly-PFOBs in different pH solutions. n. s., no significance, one-way ANOVA, Tukey's multiple comparisons test. Data are presented as mean ± standard deviation (SD) (n = 3 independent samples).

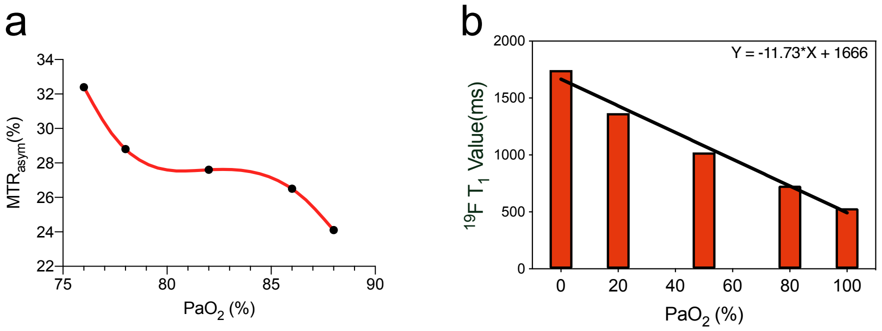

Supplementary Figure 6. ^{19}F/^{1}H-CEST signal properties of Gly-PFOBs under different oxygen partial pressure. a. CEST signal intensity of Gly-PFOBs under different oxygen partial pressure. b. ^{19}F T_1 value of Gly-PFOBs under different oxygen partial pressure. MTR_{asym}: magnetization transfer ratio asymmetry. a-b. Repeated 3 times independently with similar results.

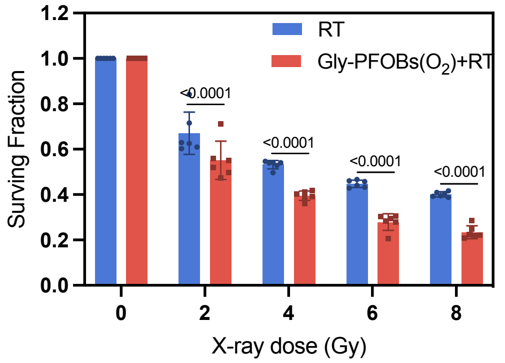

Supplementary Figure 7. Cell viability. Hypoxic cell viability results at different X-ray radiation doses (0 Gy, 2 Gy, 4 Gy, 6 Gy, or 8 Gy) in the presence of Gly-PFOBs (O_2). **** *P* < 0.0001, n = 6 independent measurements, Two-way ANOVA, Sidak's multiple comparisons test. Data are presented as mean ± standard deviation (SD). RT: radiotherapy.

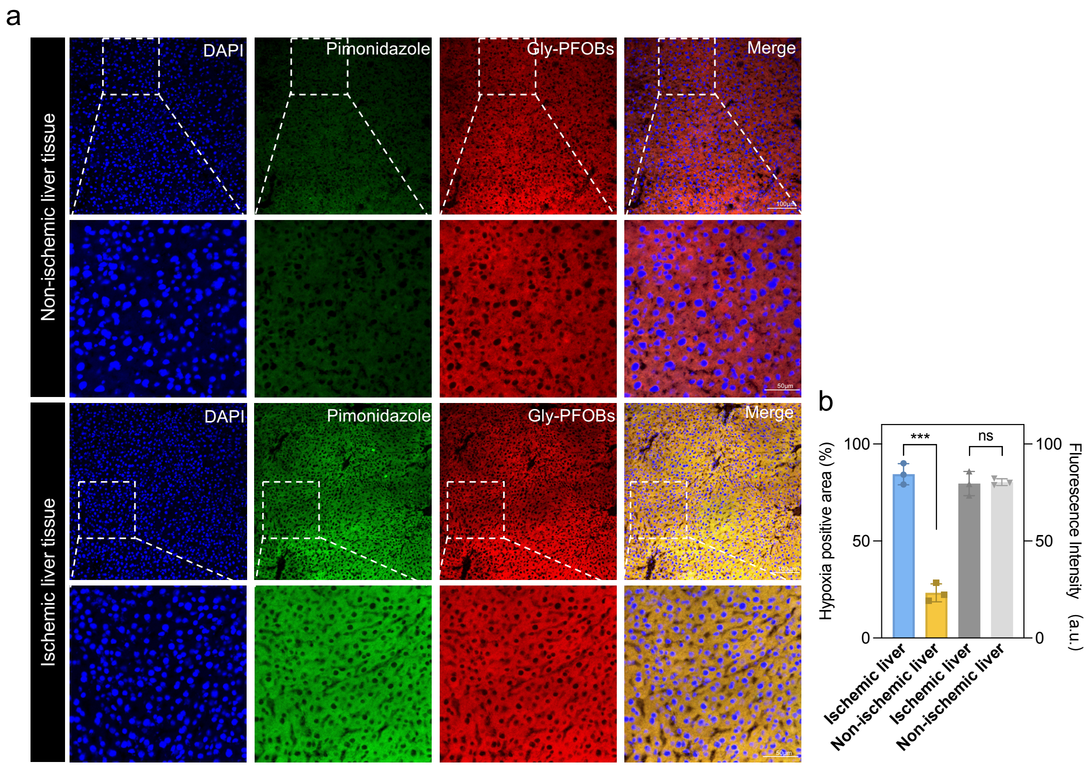

Supplementary Figure 8. Immunofluorescence results of hypoxia staining and Gly-PFOBs in liver. a. Immunofluorescence results of pimonidazole hydrochloride and rhodamine B labeled Gly-PFOBs in the ischemic and non-ischemic liver, and b. corresponding statistical results. *** *P* =0.0001, n. s., no significance. Two-tailed, unpaired t-test. n=3 independent experiments. Scale bar: 100 μm (scale bar for magnified micrograph, 50 μm). Data are presented as mean ± standard deviation (SD).

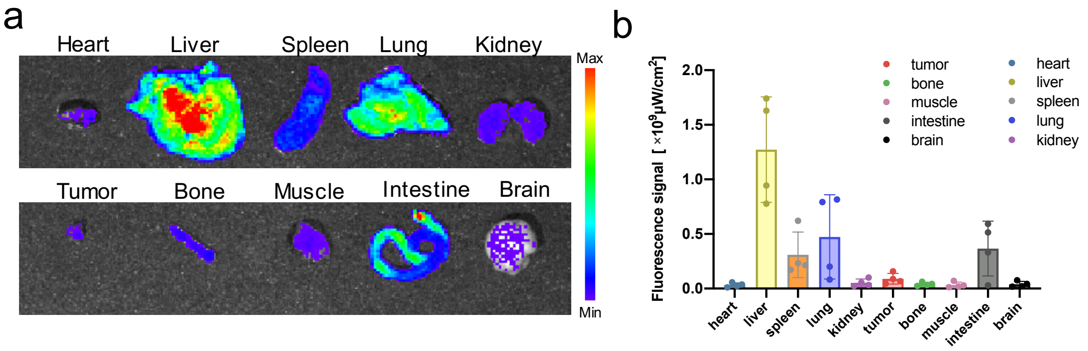

Supplementary Figure 9. Biodistribution of Gly-PFOBs in NCI-H460 tumor bearing mouse model. a. Average fluorescence intensity biodistribution percentage of tumor and major organs after intravenous injection of Gly-PFOBs, and b. corresponding *ex vivo* fluorescence analysis (n=4 mice). Data are presented as mean ± standard deviation (SD).

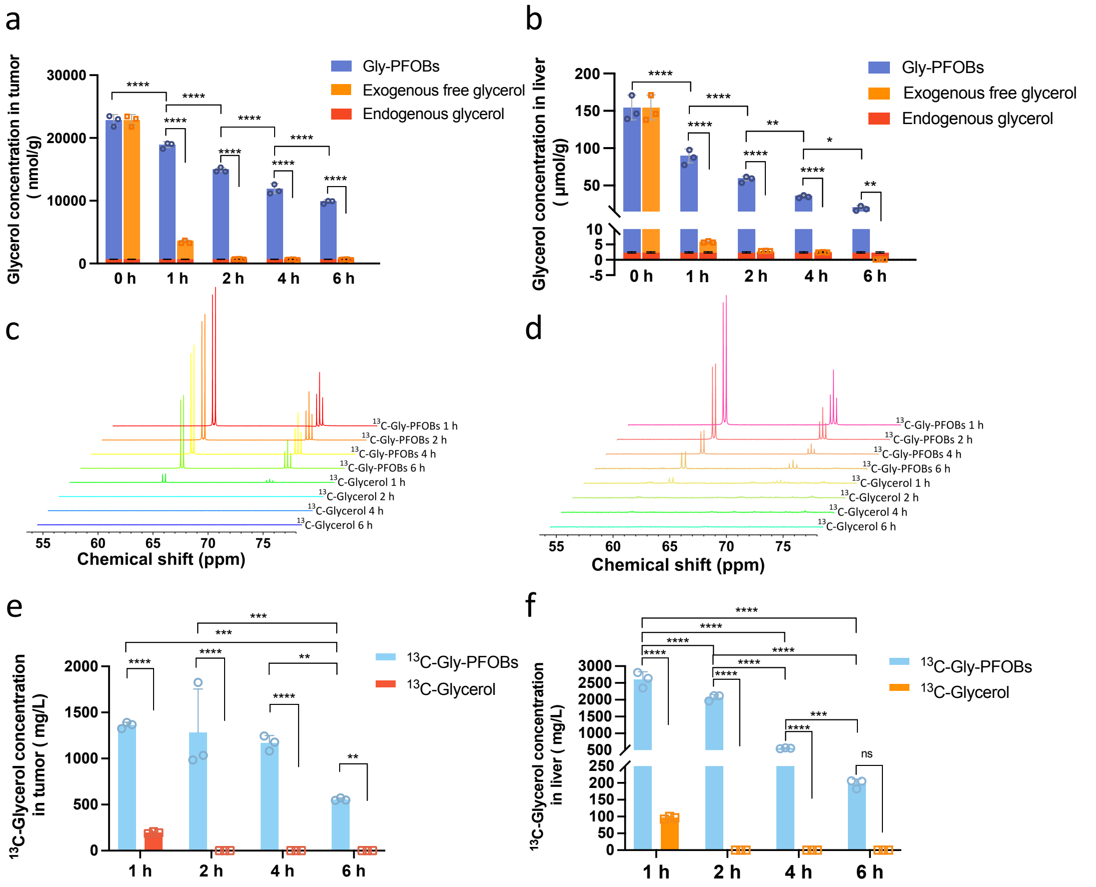

Supplementary Figure 10. The determination of *in vivo* glycerol retaining status of Gly-PFOBs in subcutaneous tumor or liver tissue. a. The glycerol concentration in tumor tissue was determined by Free Glycerol Assay Kit at different time points after intratumoral injection of Gly-PFOBs or free glycerol containing same amount of glycerol (623 mM based on glycerol, 50 μl). Meanwhile, the concentration of endogenous glycerol in tumor was also quantified for accurately reflect the exogenous glycerol concentration. **** $P < 0.0001$, n = 3 independent samples. Two-way ANOVA, Tukey's multiple comparisons test; b. The glycerol concentration in liver tissue was determined by Free Glycerol Assay Kit (Abcam, ab65337) at different time points after intravenous injection of Gly-PFOBs or free glycerol containing same amount of glycerol (623mM based on glycerol, 200 μl). The concentration of endogenous glycerol in tumor was also quantified. * P =0.0472; **P =0.0016, Gly-PFOBs 2 h *vs.* Gly-PFOBs 4 h; **P =0.0047, Gly-PFOBs 6 h *vs.* Exogenous free glycerol 6 h; **** $P < 0.0001$; n = 3 independent samples. Two-way ANOVA, Tukey's multiple comparisons test. c and e. ^{13}C-NMR (c), GC-MS (e) analysis was performed to determine the metabolic tendency or concentration of ^{13}C-glycerol in NCI-H460 tumor tissue at different time points after intratumoral injection of ^{13}C-Gly-PFOBs or free ^{13}C-glycerol containing same amount of ^{13}C-glycerol (623mM based on ^{13}C-glycerol, 50 μl), ^{13}C-glycerol inherently showed two characteristic peaks at 62.5 ppm and three characteristic peaks at 72 ppm; d and f. ^{13}C-NMR (d), GC-MS (f) analysis was used to determine the metabolic tendency or concentration of ^{13}C-glycerol in liver tissue at different time

points after intratumoral injection of ^{13}C-Gly-PFOBs or free ^{13}C-glycerol containing same amount of ^{13}C-glycerol (623 mM based on ^{13}C-glycerol, 200 μl). *P* values in (e): ** *P*=0.0027, ^{13}C-Gly-PFOBs 4 h *vs.* ^{13}C-Gly-PFOBs 6 h; *** *P*=0.0002, ^{13}C-Gly-PFOBs 1 h *vs.* ^{13}C-Gly-PFOBs 6 h; *** *P*=0.0005, ^{13}C-Gly-PFOBs 2 h *vs.* ^{13}C-Gly-PFOBs 6 h; ** *P*=0.0041, ^{13}C-Gly-PFOBs 6 h *vs.* ^{13}C-Glycerol 6 h, Two-way ANOVA, Sidak's multiple comparisons test, n = 3 independent samples. *P* values in (f): *** *P*=0.0008, ^{13}C-Gly-PFOBs 4 h *vs.* ^{13}C-Gly-PFOBs 6 h; **** *P* < 0.0001; n. s., no significance, Two-way ANOVA, Sidak's multiple comparisons test, n = 3 independent samples. Data are presented as mean ± standard deviation (SD).

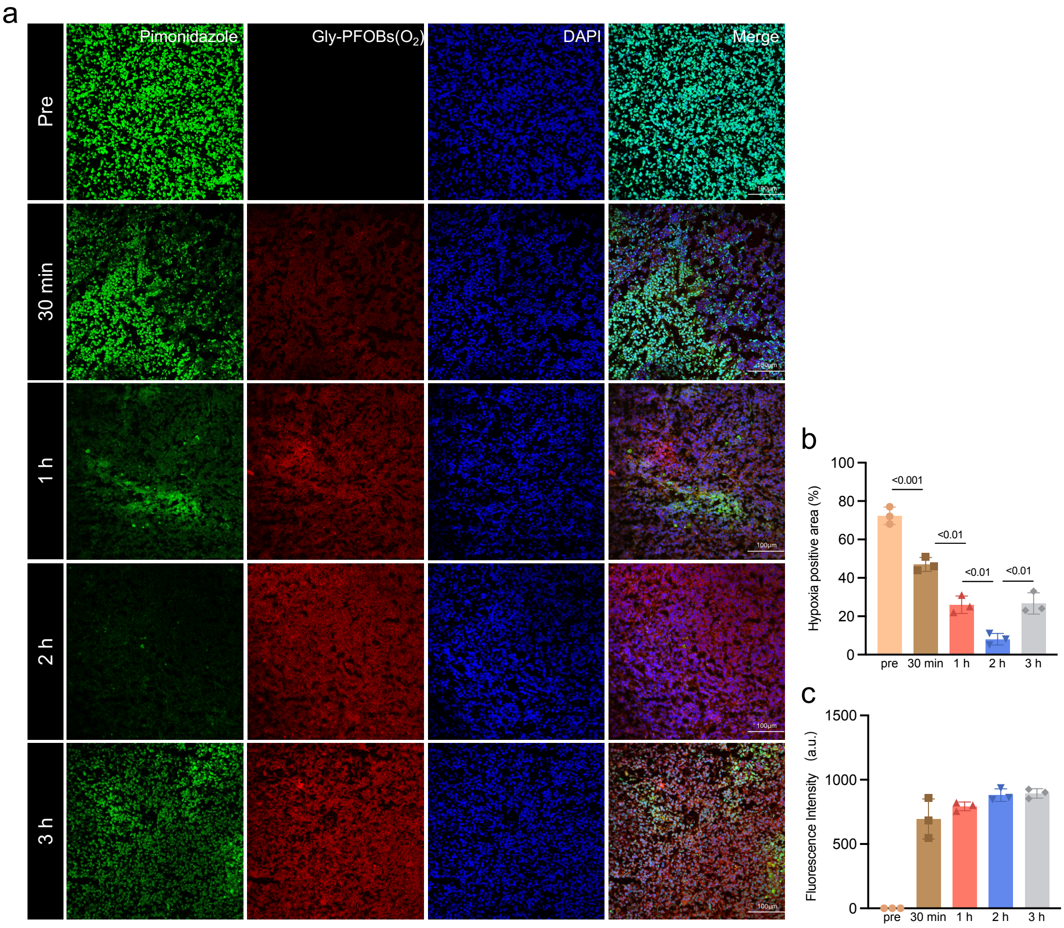

Supplementary Figure 11. Pimonidazole hydrochloride immunofluorescence staining results. **a.** Representative pimonidazole hydrochloride immunofluorescence staining results of NCI-H460 tumor tissues at different time points before and after intra-tumoral injection of rhodamine B-labeled Gly-PFOBs (O_2) b. corresponding statistical analysis of hypoxia positive area and c. fluorescence intensity of Gly-PFOBs. *** *P*=0.0002, pre *vs.* 30 min, ** *P*=0.0010, 30 min *vs.* 1 h, ** *P*=0.0013, 30 min *vs.* 3 h, ** *P*=0.0033, 1 h *vs.* 2 h, ** *P*=0.0025, 2 h *vs.* 3 h, one-way ANOVA, Tukey's multiple comparisons test, n = 3 independent experiments. Scale bar: 100 μm. Data are presented as mean ± standard deviation (SD).

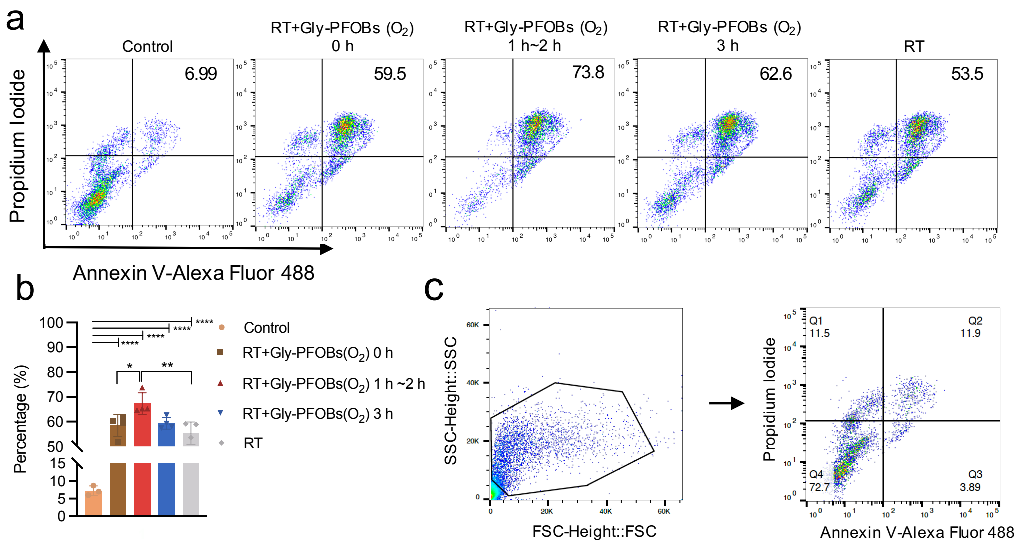

Supplementary Figure 12. **Flow cytometry analysis of cell apoptosis.** a. Determination of apoptosis level of hypoxic NCI-H460 cells co-incubated with Gly-PFOBs (O_2) probes for different duration of time (0 h, 1~2 h, and 3h) then subjected to X-ray irradiation (6 Gy). b. Corresponding statistical analysis. RT+ Gly-PFOBs 1 h~2 h *vs.* RT+ Gly-PFOBs 0 h or 3 h, $P<0.05$ ($P=0.0347$); RT+ Gly-PFOBs 1 h~2 h *vs.* RT alone, $P<0.01$ ($P=0.0039$), **** $P < 0.0001$, one-way ANOVA, Tukey's multiple comparisons test, n = 4 independent samples, n = 3 independent samples in Control group. Data are presented as mean ± standard deviation (SD). c. Representative gating strategy for the flow cytometry analysis of cell apoptosis. RT: radiotherapy.

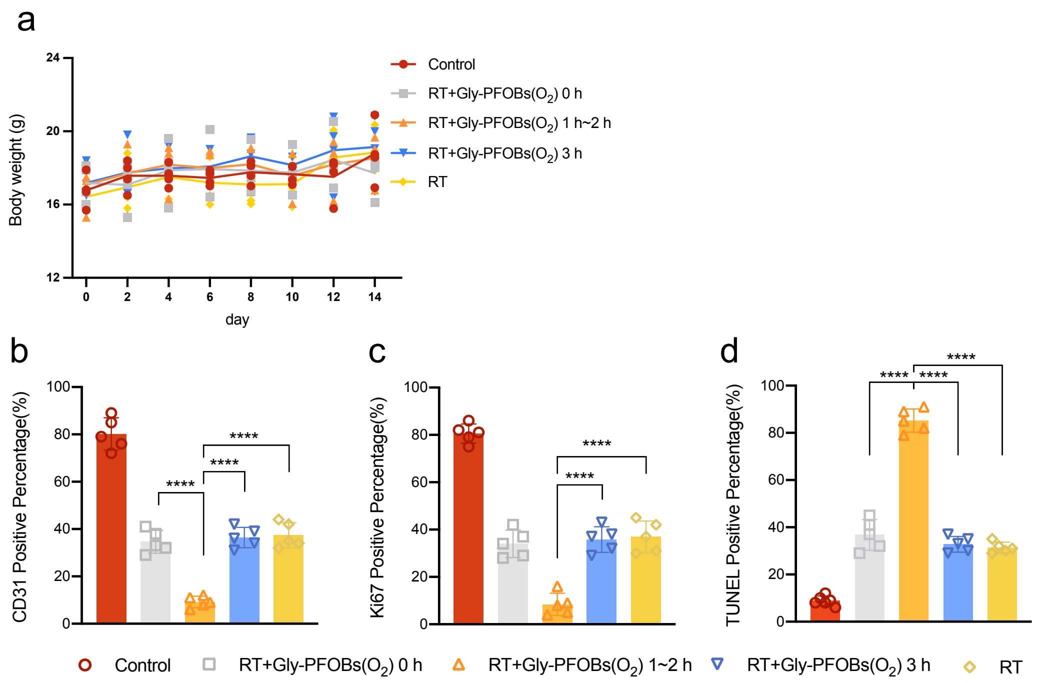

Supplementary Figure 13. Mouse body weight changes and statistical analysis of immunohistochemical staining. a. Mouse body weight changes during treatments, n = 4 mice per group. b-d. Statistical results of CD31 (b), Ki67 (c), and TUNEL (d) antigen staining in NCI-H460 subcutaneous tumor of different treatment groups. **** *P* < 0.0001, one-way ANOVA, Tukey's multiple comparisons test, n = 5 independent samples. Data are presented as mean ± standard deviation (SD). RT: radiotherapy.

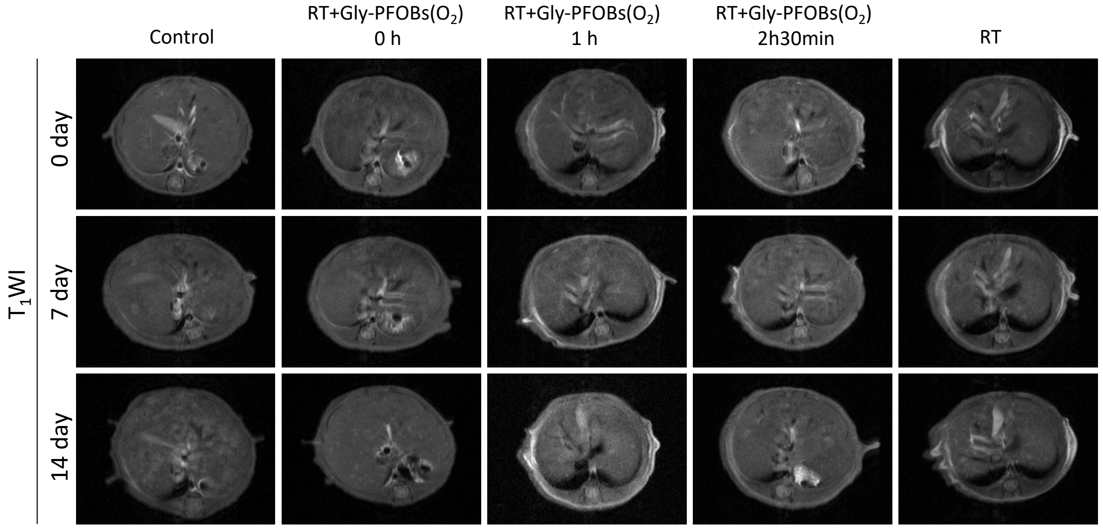

Supplementary Figure 14. T_1 weighted MR images of mice in each treatment group at assigned time points, n=3 mice per group.

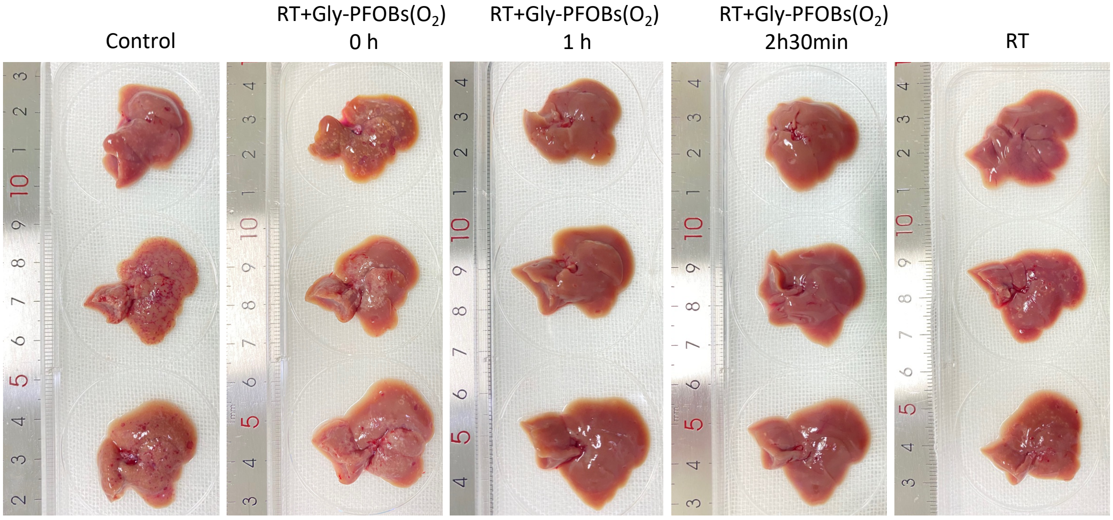

Supplementary Figure 15. **Representative livers from each treatment group.** Mice were sacrificed on day 14, and representative livers (n = 3 mice) from the treatment groups are displayed. RT: radiotherapy.

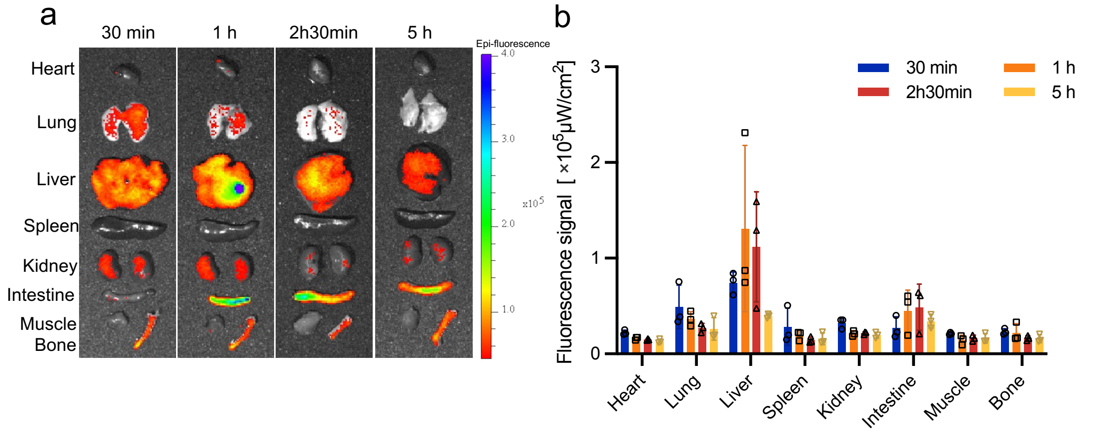

Supplementary Figure 16. **Biodistribution results of Gly-PFOBs after injection.** a. Representative IVIS imaging results of NCI-H209 SCLC liver metastases mouse model after Gly-PFOBs injection. b. Corresponding fluorescence signal analysis. Data are presented as mean ± standard deviation (SD) (n = 3 mice per group).

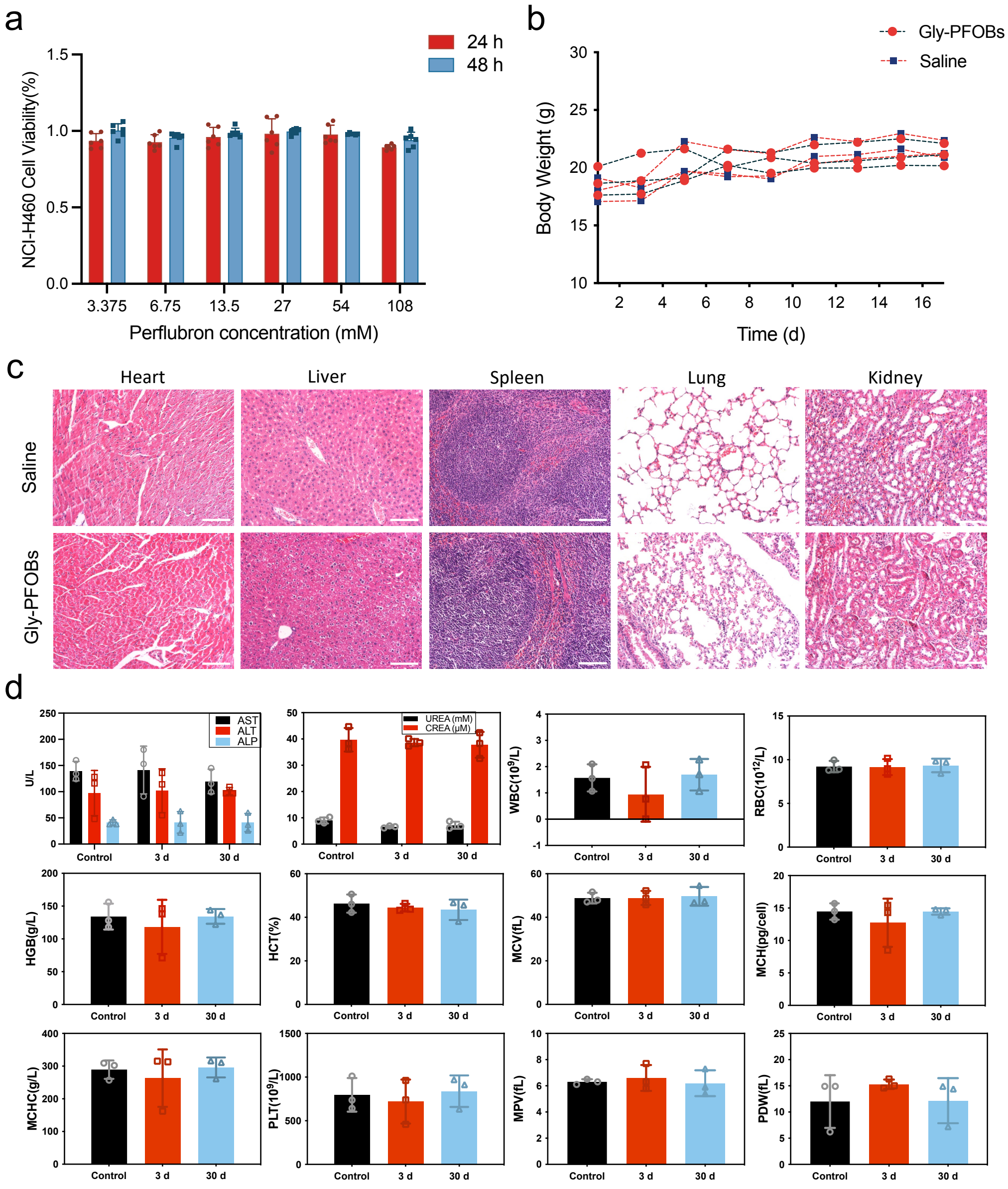

Supplementary Figure 17. Gly-PFOBs cytotoxicity and biocompatibility assessment. a. Cell viabilities of NCI-H460 cells after co-incubation with Gly-PFOBs for 24 h and 48 h, n = 6 independent measurements. Data are presented as mean ± standard deviation (SD). b. Body weight changes. Data are presented as mean ± standard deviation (SD) (n = 3 mice per group). c. H&E staining of main organ sections from BALB/c nude mice after Gly-PFOBs injection, scale bar: 100 μm, n = 3 independent samples. d. Corresponding hematological analysis of mice treated with saline or Gly-PFOBs. Data are presented as mean ± standard deviation (SD) (n = 3 independent samples). WBC: white blood cells; RBC: red blood cells; HGB: hemoglobin; HCT: hematocrit; MCV: mean corpuscular volume; MCH: mean corpuscular hemoglobin; MCHC: mean corpuscular hemoglobin concentration; PLT: platelets; MPV: mean platelet volume; PDW: platelet distribution width. Blood samples were collected for hematological analysis on day 3 and day 30 after injection, n = 3 mice per group.
