# Supplementary material for: Glycerol-weighted chemical exchange saturation transfer nanoprobes allow 19F/1H dual-modality magnetic resonance imaging-guided cancer radiotherapy
Source: Nat Commun. 2023 Oct 20;14:6644. doi: 10.1038/s41467-023-42286-3 (PMC10589257; doi:10.1038/s41467-023-42286-3)
Supplement: Supplementary file 3 — Reporting Summary [file 41467_2023_42286_MOESM3_ESM.pdf]

nature portfolio

Corresponding author(s): Xilin Sun

Last updated by author(s): Sep 15, 2023

Reporting Summary

Nature Portfolio wishes to improve the reproducibility of the work that we publish. This form provides structure for consistency and transparency in reporting. For further information on Nature Portfolio policies, see our Editorial Policies and the Editorial Policy Checklist.

Statistics

For all statistical analyses, confirm that the following items are present in the figure legend, table legend, main text, or Methods section.

n/a	Confirmed	
☐	☒	The exact sample size (*n*) for each experimental group/condition, given as a discrete number and unit of measurement
☐	☒	A statement on whether measurements were taken from distinct samples or whether the same sample was measured repeatedly
☐	☒	The statistical test(s) used AND whether they are one- or two-sided<br>*Only common tests should be described solely by name; describe more complex techniques in the Methods section.*
☒	☐	A description of all covariates tested
☒	☐	A description of any assumptions or corrections, such as tests of normality and adjustment for multiple comparisons
☐	☒	A full description of the statistical parameters including central tendency (e.g. means) or other basic estimates (e.g. regression coefficient) AND variation (e.g. standard deviation) or associated estimates of uncertainty (e.g. confidence intervals)
☐	☒	For null hypothesis testing, the test statistic (e.g. *F*, *t*, *r*) with confidence intervals, effect sizes, degrees of freedom and *P* value noted<br>*Give P values as exact values whenever suitable.*
☒	☐	For Bayesian analysis, information on the choice of priors and Markov chain Monte Carlo settings
☒	☐	For hierarchical and complex designs, identification of the appropriate level for tests and full reporting of outcomes
☒	☐	Estimates of effect sizes (e.g. Cohen's *d*, Pearson's *r*), indicating how they were calculated

Our web collection on statistics for biologists contains articles on many of the points above.

Software and code

Policy information about availability of computer code

Data collection	9.4 T MRI scanner (Bruker BioSpin 94/20 USR system, Germany), Paravision 6.0.1 system software, Microfiber optic pH transmitter equipped with pH-sensitive chemical optical pH-1 microsensor (Presens Precision Sensing GmbH, Regensburg, Germany), Agilent Technologies GC-MS system GC-7890A/MS-5977B model (Agilent Technologies, USA) ,Confocal laser scanning microscope (Nikon, Japan), Flow cytometer (Beckman Coulter, Fullerton, CA, United States), IVIS Lumina III imaging system (Caliper Life Science, USA).
Data analysis	GraphPad Prism (Prism 9; GraphPad Inc.), FlowJo 10 software (FlowJo, LLC), Image J software, Matlab (Matlab R2018b, Mathworks, Inc.)

For manuscripts utilizing custom algorithms or software that are central to the research but not yet described in published literature, software must be made available to editors and reviewers. We strongly encourage code deposition in a community repository (e.g. GitHub). See the Nature Portfolio guidelines for submitting code & software for further information.

Data

Policy information about availability of data

All manuscripts must include a data availability statement. This statement should provide the following information, where applicable:

- Accession codes, unique identifiers, or web links for publicly available datasets
- A description of any restrictions on data availability
- For clinical datasets or third party data, please ensure that the statement adheres to our policy

The authors declare that all data supporting the findings of this study are available within this Article and its Supplementary Information. Source data are provided with this paper.

Research involving human participants, their data, or biological material

Policy information about studies with human participants or human data. See also policy information about sex, gender (identity/presentation), and sexual orientation and race, ethnicity and racism.

Reporting on sex and gender	Identified in "Establishment of experimental mouse models" section in Methods.
Reporting on race, ethnicity, or other socially relevant groupings	n/a
Population characteristics	n/a
Recruitment	n/a
Ethics oversight	n/a

Note that full information on the approval of the study protocol must also be provided in the manuscript.

Field-specific reporting

Please select the one below that is the best fit for your research. If you are not sure, read the appropriate sections before making your selection.

☒ Life sciences ☐ Behavioural & social sciences ☐ Ecological, evolutionary & environmental sciences

For a reference copy of the document with all sections, see nature.com/documents/nr-reporting-summary-flat.pdf

Life sciences study design

All studies must disclose on these points even when the disclosure is negative.

Sample size	No sample size calculations were performed. Sample size was s determined to be adequate based on the consistency of measurable differences, and to ensure data's reproducibility, ≥ 3 independent replicates were performed for each experiment.
Data exclusions	No data were excluded
Replication	All studies were repeated at least three times or measured in triplicate.
Randomization	Mice were assigned randomly to standard housing and experimental groups.
Blinding	The experimenter was blind to the treatments or slide names.

Reporting for specific materials, systems and methods

We require information from authors about some types of materials, experimental systems and methods used in many studies. Here, indicate whether each material, system or method listed is relevant to your study. If you are not sure if a list item applies to your research, read the appropriate section before selecting a response.

Materials & experimental systems

n/a	Involved in the study
	☒ Antibodies
	☒ Eukaryotic cell lines
☒	Palaeontology and archaeology
	☒ Animals and other organisms
☒	Clinical data
☒	Dual use research of concern
☒	Plants

Methods

n/a	Involved in the study
☒	ChIP-seq
	☒ Flow cytometry
☒	MRI-based neuroimaging

Antibodies

Antibodies used	Ki67(Abcam, ab15580, 0.5 µg/mL) and CD31 (Abcam, ab28364, 1:50 dilution) antibodies were used to explore changes in tumor-cell proliferation, angiogenesis. Pimonidazole hydrochloride (Hypoxyprobe TM-1 plus kit, 1:200 for the FITC-MAb dilution, Hypoxyprobe Inc., Burlington, MA) was used for the determination of tumor/tissue hypoxia. The HIF-1α primary antibody (Abcam, H1alpha67, 1:100 dilution) used for cellular hypoxia evaluation.
Validation	These are all commercially available antibodies that provide validation statements on their respective websites.

Eukaryotic cell lines

Policy information about cell lines and Sex and Gender in Research

Cell line source(s)	The non-small cell lung cancer (NSCLC) cell line NCI-H460 and small cell lung cancer (SCLC) cell line NCI-H209 were purchased from ATCC.
Authentication	Cell lines have been provided and authenticated by the provider. No additional a uthentication has been performed in our labs.
Mycoplasma contamination	All cell lines were routinely tested for mycoplasma and tested negatively.
Commonly misidentified lines (See ICLAC register)	No commonly misidentified cell lines were included.

Animals and other research organisms

Policy information about studies involving animals; ARRIVE guidelines recommended for reporting animal research, and Sex and Gender in Research

Laboratory animals	1. For the antiacid treatment monitoring : n = 4 mice per group. 5-week-old female BALB/c nude mice.<br>2. For in vivo detection of the acid-base changes of ischemic liver and non-ischemic liver of mice: n = 5 mice for ischemia models, and n = 3 mice for non-ischemic models as control. 6-week-old male BALB/c mice<br>3. For the NCI-H460 NSCLC subcutaneous lung cancer model: n = 7 mice for Gly-PFOBs (O2) group and n = 6 mice for PFOBs(O2) group. 5-week-old female BALB/c nude mice.<br>4. For the NCI-H209 SCLC liver metastasis model: n=6 mice per group. 5-week-old female BALB/c nude mice.<br>5. For the biosafety experiments: n=3 mice per group. Female BALB/c mice aged 4-6 weeks.<br>All mice were purchased from Vital River Laboratory Animal Technology Co. Ltd. (certificate number: SCXK (Jing) 2016-0006, Beijing, China) and housed in a special pathogen-free (SPF) barrier facility in groups of 4 - 5 per ventilated cage with ad libitum access to food and water and kept in a 12 h light/dark cycle.
Wild animals	The study did not involve wild animals.
Reporting on sex	Female or male mice were used for different experiments. Mice of the same sex were used in same experiment.
Field-collected samples	The study did not involve samples collected from the field.
Ethics oversight	All animal procedures were carried out with the approval (permit 2022-DWSYLLCZ-67) from the Animal Ethics Committee of Harbin Medical University (Harbin, China).

Note that full information on the approval of the study protocol must also be provided in the manuscript.

Flow Cytometry

Plots

Confirm that:

- [x] The axis labels state the marker and fluorochrome used (e.g. CD4-FITC).
- [x] The axis scales are clearly visible. Include numbers along axes only for bottom left plot of group (a 'group' is an analysis of identical markers).
- [x] All plots are contour plots with outliers or pseudocolor plots.
- [x] A numerical value for number of cells or percentage (with statistics) is provided.

Methodology

Sample preparation	Cells were collected, and washed twice with cold PBS, and centrifuged at 1000 rpm for 5min at 4°C. Subsequently, cells were resuspended in 195 µL of 1× Annexin V -FITC binding buffer and incubated with 5µL of Annexin V-FITC and 10 µL of PI staining solution at room temperature for 10-20min.
Instrument	The stained cells were examined using a flow cytometer (Beckman Coulter, Fullerton, CA, United States).
Software	The results were analyzed with FlowJo 10 software (FlowJo, LLC).
Cell population abundance	For this analysis,the assay was run to a target 10,000 counts per sample
Gating strategy	Cells were first gated for live cells and get counts of live cells (SSC vs FSC) . Then Cells were gated according to the product instructions. The lower left quadrant contains AnnexinV-FITC (-) and PI (-) viable cells; the lower right quadrant, AnnexinV-FITC (+) and PI (-) early apoptotic cells; the upper right quadrant, AnnexinV-FITC (+) and PI (+) late apoptotic or necrotic cells; the upper left quadrant, AnnexinV-FITC (-) and PI (+) necrotic cells.

- [x] Tick this box to confirm that a figure exemplifying the gating strategy is provided in the Supplementary Information.
